# Cell therapy for central nervous system disorders: Current obstacles to progress

**DOI:** 10.1111/cns.13247

**Published:** 2019-10-17

**Authors:** Takao Yasuhara, Satoshi Kawauchi, Kyohei Kin, Jun Morimoto, Masahiro Kameda, Tatsuya Sasaki, Brooke Bonsack, Chase Kingsbury, Naoki Tajiri, Cesario V. Borlongan, Isao Date

**Affiliations:** ^1^ Department of Neurological Surgery Okayama University Graduate School of Medicine Okayama Japan; ^2^ Department of Neurosurgery and Brain Repair Center of Excellence for Aging and Brain Repair University of South Florida Tampa FL USA; ^3^ Department of Neurophysiology and Brain Science Nagoya City University Graduate School of Medical Sciences and Medical School Aichi Japan

**Keywords:** cell therapy, iPS cells, Parkinson's disease, stroke, traumatic brain injury

## Abstract

Cell therapy for disorders of the central nervous system has progressed to a new level of clinical application. Various clinical studies are underway for Parkinson's disease, stroke, traumatic brain injury, and various other neurological diseases. Recent biotechnological developments in cell therapy have taken advantage of the technology of induced pluripotent stem (iPS) cells. The advent of iPS cells has provided a robust stem cell donor source for neurorestoration via transplantation. Additionally, iPS cells have served as a platform for the discovery of therapeutics drugs, allowing breakthroughs in our understanding of the pathology and treatment of neurological diseases. Despite these recent advances in iPS, adult tissue‐derived mesenchymal stem cells remain the widely used donor for cell transplantation. Mesenchymal stem cells are easily isolated and amplified toward the cells' unique trophic factor‐secretion property. In this review article, the milestone achievements of cell therapy for central nervous system disorders, with equal consideration on the present translational obstacles for clinic application, are described.

## 1 INTRODUCTION

**Figure 1:**
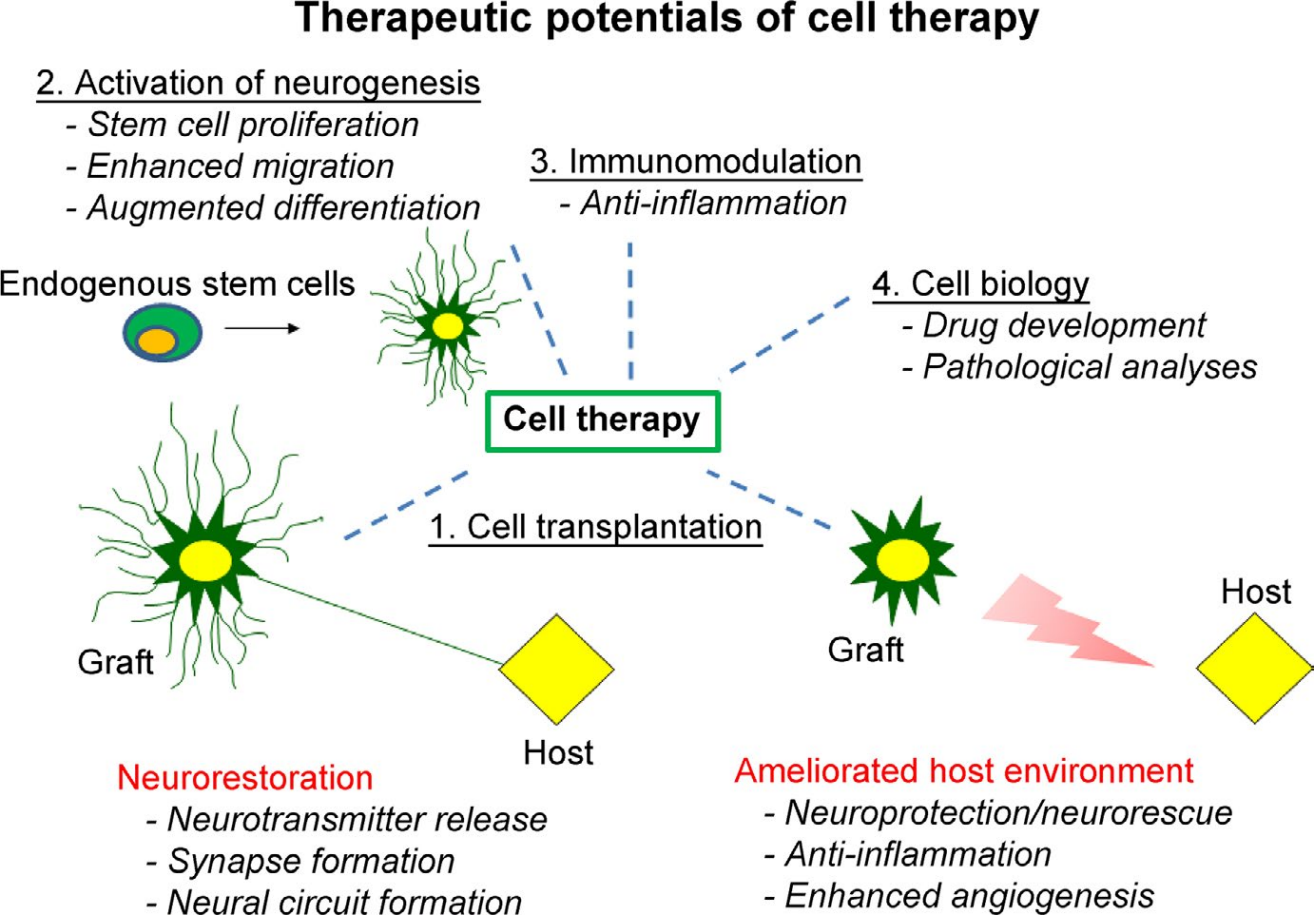
Therapeutic potentials of cell therapy are shown. 1. Neurorestoration, either by cell replacement or neural circuitry repair, is achieved by cell transplantation. 2. The activation of endogenous neurogenesis, as well as angiogenesis and vasculogenesis, provides a reservoir of proliferating new cells. 3. Systemic/local immunomodulation is one of the key factors on cell therapy. 4. Stem cell–based tools serve as drug discovery and screening of disease pathology, broadly representing another application of cell therapy

Cell therapy for central nervous system (CNS) disorders offers various therapeutic potentials (Figure 1).^1^, ^2^ First, the transplantation of exogenous cells, which include various stem/progenitor cells and differentiated cells, such as neural cells committed to specific phenotype, including astrocytes, and oligodendrocytes, is readily referred to as a form of cell therapy. Transplanted cells may function as part of a newly developed network in the host tissue^3^ or secrete several trophic factors with subsequent neuroprotective/neurorestorative capacity.^4^ Second, the activation of endogenous stem cells may serve as the foundation of the therapeutic effects of cell therapy. Several activators of this endogenous repair mechanism like exogenous stem cells, electrical/magnetic stimulation, and other stimulatory cues enhance the innate regenerative ability of the CNS.^4^, ^5^, ^6^, ^7^ Awakening of the hibernating stem cells in the hippocampus, subventricular zone, or other discreet areas in the brain; acceleration of the new cell growth in proliferative niches; enhancement of stem cells migration to the required region; and augmentation of differentiation in the targeted cells may afford powerful therapeutic effects. Third, immunomodulation may be achieved by cell therapy. Accumulating studies have demonstrated reduced immune and inflammatory responses resulting from cell therapy,^8^ indicating regulation of the immune and inflammatory reactions in the damaged or degenerating nervous system which can sequester the secondary cell death. Fourth, the development of novel drugs and screening of disease pathology via stem cell‐based tools may be viewed as one of the many applications of cell therapy.^9^

In this article, the current status of cell therapy is reviewed, with a special focus on Parkinson's disease (PD), stroke, and traumatic brain injury (TBI). The current obstacles to progress are then discussed along with possible solutions and perspectives for the future of the stem cells in the field of CNS disorders.

## 2 CELL THERAPY FOR PARKINSON'S DISEASE

Parkinson's disease is a major neurodegenerative disease caused by loss of dopaminergic neurons in the nigrostriatal system characterized by resting tremor, rigidity, akinesia, and postural reflex disturbance as representative symptoms. Dopamine replacement therapy^10^ in conjunction with other medications and surgical procedures such as subthalamic nucleus deep brain stimulation^11^ and thalamotomy are established treatments for PD. However, current treatments focus only on the suppression of symptoms, and there is no treatment capable of stopping or improving the pathological condition itself. Thus, regenerative medicine, and in particular cell therapy, has attracted the attention of many scientists, doctors, and patients, because of its potential for reinnervation of the neuronal network and neurorestoration, allowing disease‐modifying instead of palliative outcomes.^1^

Since Perlow and colleagues first demonstrated in 1979 that brain tissue grafts of dopaminergic neurons ameliorate behavioral abnormalities in the rat model of PD,^12^ several investigations have been pursued to develop cell therapy into a safe and effective therapeutic strategy for PD in both basic and clinical arenas. Based on overwhelming preclinical experiments demonstrating improved behavioral and histological deficits in transplanted parkinsonian animals, two clinical studies of fetal nigral cell transplantation in PD patients were reported.^13^, ^14^ For the next decade, fetal nigral cell transplantation was performed in the United States and Europe. However, after Freed and coworkers reported the limited efficacy of fetal nigral cell transplantation,^15^ the positive momentum of this type of cell transplantation diminished. Recently, the TRANSEURO trial, a European Union‐funded multicenter clinical trial of fetal nigral cell transplantation, has invited renewed enthusiasm in cell therapy for PD due to positive clinical outcomes for selected patients.^16^, ^17^

Other than fetal nigral cell transplantation, autologous dopaminergic cells, embryonic stem cells, neural stem cells, mesenchymal stem cells (MSCs), and other cells have been considered good transplantable cell candidates. A literature search in ClinicalTrials.gov, using the key terms “Parkinson's disease” and “transplantation,” revealed 29 trials (as of November 2018), although some trials are labeled as terminated, while the details of the other trials are unknown. The cell sources for these trials involved fetal nigral cells (6), embryonic stem cells (1), MSCs (6), neural stem cells (5), induced pluripotent stem cells (iPS cells) (1), and others (10). iPS cells may represent unique transplantable features compared with the other cell sources. In 2006, Takahashi and Yamanaka established iPS cells from mouse embryonic and adult fibroblast cell culture by adding four factors: Oct3/4, Sox2, c‐Myc, and Klf4.^18^ This discovery is widely heralded as pivotal to the progress of cell therapy toward clinical applications, with many subsequent studies detailing the viability and reproducibility of iPS cells, coupled with therapeutic efficacy. The first clinical application of iPS cells in Japan was performed by using autologous iPS cells, which were differentiated into mature retinal pigment epithelial cells, for patients with macular degeneration.^19^ In this study, no significant adverse events were noted at 1 year post‐transplantation. For PD, several teams explored a therapeutic strategy using autologous iPS cell–derived neurons.^20^ One of the merits of autologous cells is they circumvent ethical issues associated with fetal and embryonic cells. Additionally, such same donor‐recipient of the stem cells may avoid immunological problems. However, the logistics in establishing a homogenous population of iPS cells with phenotypic profile and functionality of a dopaminergic neuron remain a challenge toward clinical application. Moreover, the harvest of iPS cells from PD patients may present with cells containing the disease pathology, thus may succumb to accelerated neurodegeneration as opposed to iPS cells derived from healthy donors. To overcome these problems related to autologous iPS cells, nonautologous iPS cell–derived cells may be an alternative transplantable source; however, immune reactions are likely to arise with the use of mismatched donor cells. Ensuring the quality of the phenotype and function of the differentiated iPS cells should be approached in tandem with the safety of both autologous and nonautologous iPS cells. A recent study demonstrated that iPS cells from several specified human leukocyte antigen (HLA)‐homozygous donors display no detectable immune rejection, suggesting their potential as safe and effective donor cells.^21^ In Japan, specified allogeneic iPS cell–derived dopamine neurons have been generated under high‐safety protocols.^22^ Very recently, a Japanese team successfully implanted for the first time dopamine neurons derived from allogeneic iPS cells into a PD patient (https://www.nature.com/articles/d41586-018-07407-9). An international organization focusing on safety of cell transplantation, GForce‐PD, is actively monitoring these trials (http://www.gforce-pd.com/).^23^ Stem cell transplantation with the use of iPS cells for PD patients has now reached another significant milestone, reminiscent of the excitement in the 80 seconds and 90 seconds when fetal cells were first implanted in PD animals and patients.

## 3 CELL THERAPY FOR STROKE

Stroke is currently one of the most examined CNS disorders for cell therapy. A search of ClinicalTrials.gov for “stroke,” “brain,” and “transplantation” generated 34 studies. In almost 70% of the studies, MSCs or related cells were used. In stroke, multiple cells, including neurons, astrocytes, oligodendrocytes, and other cells, succumb to abrupt death following the stroke onset with many more cells exposed to progressive degeneration due to subsequent secondary damage.^24^ The disease progression is completely different from that of PD, which is characterized primarily by dopaminergic neuronal degeneration. The true restoration for stroke is different from that for PD in that the regeneration for many types of cells, coined neurovascular unit, is required. Recognition of key cell‐to‐cell interactions as a network has been acknowledged in stroke degeneration and regeneration. Considering the multiplicity of cell types and their functions affected by stroke, robust and long‐lasting regeneration for stroke appears very difficult under existing circumstances. The goal to arrest cell death in the neurovascular unit is paramount to devising cell‐based regenerative medicine. To this end, MSCs have shown promise in regenerating the neurovascular unit. The advantages of MSCs are (a) rapid isolation from bone marrow, (b) efficient amplification in culture, (c) easy maintenance in culture, (d) suitability for autologous transplantation even in the acute phase of stroke, and (e) solid neurotrophic effects.^25^ The therapeutic effects of MSCs may be mediated by many regenerative mechanisms, which include angiogenesis, anit‐inflammation, antiapoptosis, neurogenesis with subsequent cell migration, and differentiation.^26^ Due to their long‐track record of safety in hematologic diseases and a bulk of preclinical stroke studies demonstrating safety and efficacy, MSCs have been the focus of many clinical studies. Cell delivery of MSCs entails intracerebral, intra‐arterial, or intravenous route.^27^ An intracerebral approach may be the most effective, but also the most invasive, while an intravenous approach may be the least invasive but with the fewest cells reaching the targeted area in the brain. The intra‐arterial approach may lie somewhere between the two cell delivery routes. In 2005, a phase I study with an intravenous administration of autologous MSCs was first performed for patients with ischemic stroke.^28^ In 2014, the first phase II clinical trial involving intravenous administration with allogeneic MSCs for patients with ischemic stroke was published.^29^ In this study, the therapeutic effects of intravenous administration of allogeneic MSCs were reported for patients with ischemic stroke in the subacute phase. In a study using intra‐arterial allogeneic MSC administration, 40% of stroke patients who receive transplantation exhibited good clinical outcomes within 3‐7 days after onset.^29^ The administration route and the timing for stroke patients may require more optimization because of the differences in stroke severity across patients. In Japan, several clinical studies are ongoing. Shichinohe and teammates reported their protocol of intracerebral administration of autologous MSCs in the subacute phase of stroke.^30^ They proposed to use cells labeled with superparamagnetic iron oxide for cell tracking, which may reveal the distribution of the transplanted cells over time.

## 4 CELL THERAPY FOR TRAUMATIC BRAIN INJURY

Traumatic brain injury is a common neurological disorder caused by physical trauma to the brain that affects all ages and has long‐lasting effects. To date, there is no effective drug treatment for TBI, and patients are often left depending on rehabilitation and symptom management, which had limited effectiveness.^31^ Ideally, an effective treatment would be one that offers an extended therapeutic window and reduces secondary cell death progression. Furthermore, to combat the complex secondary cell degradation, it is necessary to have a treatment that acts through diverse mechanisms in order to successfully translate to the clinic. This presents an important research prospect for regenerative medicine. To this end, there is an emerging field of study that posits stem cell transplants as an ideal option because they can provide increased growth factors levels, ameliorate neuroinflammation, inhibit apoptotic pathways, offer a wide therapeutic window, and apply to a larger number of patients.

Previously, TBI was classified as an acute injury due to the lack of understanding of the chronic functional deficits and pathological symptoms.^32^, ^33^ However, TBI is now considered a chronic disease because of the secondary inflammatory response that accompanies the initial insult.^32^, ^33^ Currently, there remains a dearth of clinical trials for TBI therapies, with most of the ones that do exist relying entirely on rehabilitation treatment.^34^, ^35^, ^36^, ^37^, ^38^, ^39^ Since TBI is followed by a chronic neurodegeneration phase, more emphasis has been placed recently on neuroregenerative medicine rather than neuroprotective medicine, which may be effective only in the acute phase of the injury.^40^, ^41^ Therefore, stem cell therapy is a clear option and, indeed, has a history of inducing robust functional recovery in clinical and laboratory settings, including those conducted on TBI models.^42^, ^43^, ^44^, ^45^ Despite these merits, translating stem cell therapy to the clinic has proven to be a difficult task.^46^ Further investigation is warranted in order to find the mechanism by which stem cells provide regenerative properties in TBI brains, in addition to ideal treatment regimes.

Determining the optimal stem cell source is likewise essential to ensure ethical clearance and graft integrity, and also to assure the replicability and the validity observed in laboratory settings. More research is needed to identify the optimal cell line that can be harvested regularly and safely delivered to the patients. Even so, stem cells offer neuroprotective effects through multipronged pathways, such as those that support neurogenesis and reduce neuroinflammation,^47^, ^48^, ^49^ while simultaneously increasing vasculogenesis and angiogenesis.^50^, ^51^, ^52^ Notably, the cytotoxic environment that exacerbates and is produced by the secondary inflammatory response may also be responsible for the low graft survival rates that have been observed in the TBI brain.^53^ Nonetheless, functional recovery still prevails, implying that another factor besides graft survival and direct replacement may engender some of the recorded benefits. In addition, reducing the toxicity of the microenvironment may lead to increased graft survival and amplified neuroprotective bystander effects is an promising therapeutic approach for future studies with the ultimate goal of translating stem cell therapy for use in the clinic.

At first, stem cell transplants were intuitively thought to function in the CNS by replacing damaged neural cells with new, viable cells in a one‐to‐one fashion. As mentioned above, it has been observed that stem cell transplantation directly into the damaged tissue leads to poor graft survival. Nevertheless, functional recovery and reduced neural death have still been observed despite the poor retention.^54^ Therefore, more complex mechanisms must be responsible for stem cells' therapeutic potential, instead of their previously theorized long‐term survival and differentiation.

In response, other mechanisms of action have been proposed to clarify this seeming incongruity. That transplanted stem cells may secrete neurotrophic factors represents the first of these. These neurotrophic factors generally confer therapeutic effects via their activation of cell survival pathways, yet TBI usually entails a decrease in their expression. In this respect, treatment intended to rescue these expression levels may circumvent the TBI‐induced apoptosis in the peri‐impact area.^55^, ^56^ While preclinical studies of stroke have revealed that administering stand‐alone glial cell line–derived neurotrophic factor (GDNF),^57^ brain‐derived neurotrophic factor (BDNF),^58^ vascular endothelial growth factor (VEGF),^56^ stem cell factor (SCF),^59^ or stromal cell–derived factor (SDF)‐1α^60^ may produce improvements on neurological outcomes, various complications limit the likelihood of clinical success. These complications entail determining the correct types, dosages, and timing of these factors and thus represent a significant challenge. Inappropriately high doses may do more harm than good; for example, drug‐induced overproduction of BDNF has been documented to trigger epileptic seizures.^61^ To this end, stem cells possess an innate ability to respond to the minute‐to‐minute status of their environment and adjust the levels of their secreted neurotrophic factors accordingly.^55^ By affording an in situ source for these factors, transplanted stem cells may reduce inflammation and increase cell survival.

Another mechanism of action whereby stem cell transplants may confer indirect therapeutic effects is their activation and amplification of natural neuroprotective responses that may otherwise remain latent or impotent. That adult brains are unable to regenerate neurons was invalidated by recent evidence of endogenous stem cells housed in the neurogenic niches: the subgranular zone (SGZ) of the dentate gyrus and the subventricular zone (SVZ) of the lateral ventricles.^62^, ^63^ Therefore, under the right conditions, these cells may promote neurogenesis, which could be particularly useful as a therapeutic tactic to combat neurological insults.^64^ However, the restrained capacity of these cells to commit to a neuronal lineage, differentiate, and mobilize to the impact region from the neurogenic niches has stymied this therapeutic avenue.^65^ Until recently, endogenous stem cells' largely insufficient prevention or reduction of pathological pathway‐induced cell death progression has seemed to be a dead end. Yet, exogenous stem cell transplants have unveiled a new path to facilitate the lengthy migration of these endogenous stem cells and thus allow their effects to be therapeutically relevant. A frontal cortex controlled cortical impact (CCI) rodent model of TBI demonstrates enhanced recovery in the group treated with intracranially delivered MSCs compared to the vehicle group^66^; importantly, according to past evidence, the frontal cortex was considered too distant to be accessible by most endogenous stem cells. Upon immunohistochemical analysis and laser capture microdissection, however, the observation of a MSC‐paved “biobridge” linking the neurogenic niche and the damaged frontal cortex posits a potential mechanism by which transplanted stem cells may recruit the endogenous stem cells to injury site.^66^ This novel theory of a biobridge has not been observed in any procedure other than stem cell transplantation and is believed to mediate the neuroprotective and neuroregenerative actions of endogenous stem cells. Thus, transplanted stem cells and endogenous stem cells may act together to protect and restore the TBI‐damaged brain.

The transplanted stem cells' secretome, which constitutes the sum of their secreted factors, has been proposed as a fourth mechanism by which stem cell transplants may grant indirect therapeutic effects after TBI. In addition to the various neurotrophic factors produced from the corpus of the cell, stem cells may also emit exosomes and microvesicles, which, in turn, may release chemokines, cytokines, long noncoding RNA (lncRNA), microRNA, and growth factors such as VEGF.^67^ That this secretome may confer therapeutic effects on lung, cardiovascular, liver, and kidney disease has been supported by improvements observed after treatment of isolated microvesicles and exosomes obtained from multipotent MSCs.^67^ Furthermore, in a rodent model of TBI, the groups injected with MSC secretome display lower levels of brain damage volume and apoptosis, and exhibit higher levels of regenerated neurons and improved scores on cognitive and motor functional assessments when compared to the control group.^68^ Thus, stem cell transplants present multiple treatment strategies that may be harnessed to improve the current standard of care for TBI patients.

## 5 CELL THERAPY FOR OTHER CENTRAL NERVOUS SYSTEM DISORDERS

Cell therapy for CNS disorders covers a broad range of pathological conditions, such as spinal cord injury,^69^ amyotrophic lateral sclerosis,^70^ Huntington's disease,^71^ and cerebral palsy.^72^ Psychiatric diseases are one of the targets of cell therapy, and depression is a common disease that is one of the major causes of disability. Although pharmacological treatment has improved, only roughly 50% of depressed patients respond to this method.^73^ Recently, we reported on the therapeutic potential of encapsulated mesenchymal stem cells for a depression model of rats.^74^ The treatment improved depression‐like behavior with enhancement of the endogenous neurogenesis in the hippocampus and the subventricular zone. The activation of signaling with various trophic factors, including VEGF, BDNF, ciliary neurotrophic factor, and fibroblast growth factor 2, was involved in the therapeutic effects. Thus, cell therapy might offer hope for psychiatric disorders.

## 6 CURRENT OBSTACLES THAT HINDER LABORATORY‐TO‐CLINIC PROGRESS

Cell therapy for CNS disorders has generally advanced toward clinical application, but technical and logistical problems perspectives remain, which are discussed below.

### 6.1 Enhancement of the therapeutic effects of cell therapy

Several cells are considered promising candidates for cell therapy. However, optimizing the therapeutic outcomes of transplanted cells is warranted. Rehabilitation, certain pharmacologic agents, and electrical/magnetic stimulation may serve as adjunct treatments that may enhance cell therapy by providing important cues for stem cell differentiation, migration, or synapse network formation, which are critical indices of CNS regeneration. In a mouse model of chronic spinal cord injury, improvement in allodynia and hyperalgesia was observed animals that received neural stem/progenitor cell transplantation with rehabilitation.^75^ Electrical stimulation was also shown to trigger the migration of intracerebrally transplanted mesenchymal stromal cells in experimentally stroke rats through SDF‐1α signaling.^7^ Long‐term potentiation enhances neurogenesis in the hippocampus.^76^, ^77^ Additionally, electrical stimulation improves synapse formation, altogether facilitating the therapeutic effects of cell therapy and suggesting the potential of combined approaches.

### 6.2 Safety of cell therapy

Autologous patient‐derived cells may circumvent logistical and ethical problems. However, genetic engineering or reprogramming of these adult cells to amplify stemness can lead to uncontrolled proliferative capacity. On the other hand, if these patient‐derived cells are left unmanipulated the disease pathology, including genetic abnormality may limit their viability and therapeutic potential. Accordingly, careful manipulation of these cells is needed to ensure stemness while regulating their proliferation. Alternatively, the use of healthy donors for allogeneic may avoid the disease phenotype of cell source, but these cells run the risk of immune rejection. To this end, there is a need to eliminate uncertain factors for cell differentiation, remove cells at risks for tumorigenesis, and purify the differentiated cells regardless of donor origin. Even after thorough testing, failsafe procedures should be confirmed by various methods in clinical settings.^78^

### 6.3 Concerns about iPS cells

iPS cells as autologous stem cells have become an attractive cell source for transplantation. For transition to clinical application, reduction in time to convert these cells into the desired cell phenotype, and the cost associated with such cell culture to establish, maintain and use of iPS cells for therapeutic purpose are key enabling studies.^79^ Inter‐clonal differences, nonhomogeneity of differentiation, and varied genetic backgrounds represent additional technical issues when priming iPS cell–derived differentiated neurons, oligodendrocytes, or astrocytes as cell products for CNS disorders. Gene expression of dopaminergic neurons derived from iPS cells of PD patients was reported to be significantly different from that of primary dopaminergic neurons.^80^ Beyond replacement of functional cells for patients with CNS disorders, iPS cells have broad potentials for establishing disease models that will be valuable for recapitulating pathological conditions and subsequent drug development. In the end, the genetic alteration, reaction to drugs, and age of the cells limit the use of autologous patient‐derived iPS cells compared to allogeneic healthy donor‐derived iPS cells. Alternatively, the direct conversion or trans‐differentiation from fibroblasts into neurons while skipping the iPS cell states may circumvent these technical problems.^81^

### 6.4 Evaluating the potential of cell therapy

Different approaches for testing the safety and efficacy of cell therapy have been evaluated in clinical trials. The quality and well‐defined characteristics of the stem cells are basic criteria that need to be ascertained prior to any clinical application. Equally important is the rigorous clinical trial design in order to ensure unbiased, reproducible, and valid outcomes. The primary outcomes for the trials should be carefully considered, together with the dose, route, and timing of cell administration. Differences in clinical trial designs have made difficult comparing the outcomes between studies. In determining efficacy, ample consideration should also be given to evaluating safety of cell therapy. Minimally invasive procedures, such as intravenous and intra‐arterial routes, may not present much safety issues related to the injection site as opposed to more invasive administrations (eg, intra‐thecal, and intracerebral) which should be performed with much greater care by a skilled clinician. A center of excellence for regenerative medicine is likely to facilitate the regulated conduct of a clinical trial, allowing rigorous evaluation of the safety and efficacy of cell therapy.

### 6.5 Protection of patients' rights

The care of human research subjects is also extremely important. Complications arising from the procedure itself, the transplanted cells, and cell‐associated factors need to be closely monitored to protect the patients participating in clinical trials, especially in the case of invasive cell procedures. A safety monitoring board should be tasked to perform such adverse outcome monitoring not only during the acute post‐transplantation period but also throughout long‐term post‐transplantation period. The control and maintenance of privacy, including genetic information, should also be considered. Legal systems, the insurance regimen, and the institutional review board should be reconsidered with a focus on the education for both doctors and patients in response to the entry of cell therapy from small scale to a larger clinical trials.

## 7 CONCLUSION

Cell therapy has progressed considerably, and further advances are being made. While the current status of cell therapy for CNS disorders is promising, we need to overcome various obstacles in the near future.

## CONFLICT OF INTEREST

The authors declare no conflict of interest.
